# Malaria therapy for neurosyphilis at Mont Park Hospital for the insane in Australia, 1927–1928

**DOI:** 10.1177/0957154X251403921

**Published:** 2025-12-30

**Authors:** Alison Clayton

**Affiliations:** 1 University of Melbourne, Parkville, VIC, Australia

**Keywords:** malaria therapy, general paralysis of the insane, neurosyphilis, Reginald Ellery, history of psychiatry

## Abstract

A century ago, malaria therapy for general paralysis of the insane was a celebrated Nobel Prize-winning psychiatric treatment widely used across the globe. However, in recent years, its legacy has come under increasing scrutiny, with some historians and physicians questioning the claims of it being an effective treatment. This paper, utilizing published accounts and medical record archives, describes malaria therapy for neurosyphilis as conducted by psychiatrist, Reginald Ellery at Victoria’s Mont Park Hospital for the Insane, 1927–1928. It also evaluates Ellery’s claims about malaria therapy’s effectiveness. This research delineates several clinical and research-related factors – such as diagnostic drift, adjunct interventions, and healthy-patient selection – that likely contributed to an overestimation of malaria therapy’s effectiveness. Additionally, it demonstrates that patient outcomes as reported in the medical records are notably less positive than described by Ellery in his published accounts. Consequentially, the findings of this study do not support the claims of malaria being an effective treatment for neurosyphilis.

## Introduction

At the turn of the 20th century, general paralysis of the insane (GPI), a severe form of neurosyphilis, was a deadly and dreaded illness, which could account for up to twenty percent of male admissions to the mental asylums (Davis, 2008: 15). In 1917, Julius Wagner-Jauregg, a Viennese psychiatrist, invented malaria fever therapy as a treatment for GPI, for which he was awarded the 1927 Nobel Prize (Wagner-Jauregg, 1927). Wagner-Jauregg (1927) claimed full remissions in 50 to 85% (dependent on stage of illness) of his malaria-treated GPI patients, which he described as “far exceeding” results obtained by other methods. Malaria therapy was a popular treatment and widely used across the globe until the 1950s, at which time it was replaced by penicillin treatment. The extant historical literature emphasizes the pivotal role that GPI and malaria therapy played in the development of 20th-century psychiatry and its quest to identify the biological origins of mental illnesses and for heroic somatic cures (for example, see Harrington, 2008: 60; Scull, 2015: 302, 308; Shorter, 1997: 194). In recent years, a rich historiography of malaria therapy has emerged, examining it from various perspectives and grounded in research based on the local medical record archives (Braslow, 1997: 55–71; Clayton, 2022; Daey Ouwens et al., 2017; Davis, 2008; Gambino, 2015; Hurn, 1998: 192–259; Kragh, 2010).

Malaria therapy’s status as an effective treatment is a topic of debate in both the medical and historical literature.^1^ Some still describe it as having been a highly effective treatment (Brown, 2000; Kaplan, 2010; Whitrow, 1994; Sadowsky, 2021: 106, 210; Shorter, 1997: 194, 379). However, in recent years, this claim has come under increasing scrutiny. Critics note that the era’s observational studies were limited by poorly described patient characteristics, variable diagnostic criteria, and inadequate follow-up. Importantly, the natural course of GPI included remarkable spontaneous remissions, making it hard to judge the impact of any treatment (Austin et al., 1992; Frankenburg and Baldessarini, 2008; Scull, 2015: 301–302). It is important to highlight that some malaria-era clinicians, in a manner that prefigured these types of current-day evidence-based medicine precepts, questioned the lavish claims made about its reported benefits (Bolton, 1929; Reid, 1932). Furthermore, the difference between the optimistic rhetoric of the published accounts compared to the pessimistic nature of the medical records accounts has been observed (Clayton, 2022; Davis; 2008: 243).

In a previous publication, I reported on psychiatrist Reginald Ellery’s introduction of malaria therapy into Victoria at the Sunbury Hospital for the Insane from 1925 to 1926 (Clayton, 2022). Ellery (1927) described that in contrast to the previously hopeless nature of GPI treatment, 70% of the Sunbury hospital GPI patients he treated with malaria had improved, with 30% of being “apparently cured”. By 1929, he was claiming that whereas a few years previously a diagnosis of GPI had been a death warrant, remissions could now be produced in a large percentage of patients “almost at will”, thus, “some of the blackest clouds have passed out of the psychiatric sky” (EM/SLV, 1929a: 3).

However, my previous research (Clayton, 2022) revealed that various clinical and research factors, including the selection of healthier patients and diagnostic inaccuracies, likely led to Ellery overestimating malaria therapy’s effectiveness. Furthermore, my analysis of the medical record archives indicated a notably less favourable assessment of malaria therapy than that presented in Ellery’s published accounts. However, Clayton (2022) focused on Ellery’s preliminary trials of malaria therapy that involved only 10 GPI patients. A critical question is whether similar findings can be observed in Ellery’s later practice of malaria therapy with a larger cohort of patients at Victoria’s Mont Park Hospital for the Insane (forthwith Mont Park).

In this current paper, I have two primary objectives. First, drawing on published accounts and the medical records archive, I will provide a detailed historical account of Ellery’s malaria therapy practice at Mont Park. Second, I will utilize insights from this local-setting context to build upon the existing scholarship that critically assesses the purported effectiveness of malaria therapy. To begin, I provide a comprehensive overview of Mont Park Hospital, Reginald Ellery, and Mont Park’s malaria fever clinic during the years 1927–1928. Following this, I critically evaluate five pertinent issues that are essential for comprehending the perceived effectiveness of malaria therapy. These issues encompass evolving diagnostic practices; the Victorian Lunacy Department’s dependence on an obsolete and inaccurate Wassermann test technique; the emergence of a novel patient category – the voluntary neurosyphilis patient; the use of adjunctive interventions alongside malaria therapy; and the inconsistencies between the medical records’ accounts and Ellery’s published narratives. Lastly, I provide an overview of the progress and eventual decline of malaria therapy at Mont Park from 1930 to 1950.

## Methodology

The research presented in this paper has been undertaken as part of a University of Melbourne PhD research project. University of Melbourne human research ethics committee approval (ID: 2057066.1) was obtained. The Public Records Office of Victoria (PROV), that holds the medical record archives of Victoria’s Lunacy Department, also gave approval for this research. I identified patients diagnosed with neurosyphilis at Victoria’s Mont Park Hospital for the Insane, 1927–1928, through cross-checking data contained in multiple medical record archival documents. Another key source, held at the State Library of Victoria, is a document written by Ellery and titled “Statistics of Cases Treated by Malaria 1928” (henceforth “Ellery’s List”) (EM/SLV, 1929b). In his list, Ellery documented the name, date of malaria treatment, and outcome (as of June 1929)^2^ of the 90 neurosyphilis patients he treated from November 1927 through until December 1928. He classified patient outcomes as discharged, improved, unimproved or dead, and then calculated the percentages for these outcomes, which he then included in his published accounts. I was able to locate medical record archive information^3^ for 98% of the patients described in Ellery’s List. The narrow time frame of this current study has enabled me to access medical record data on almost all the neurosyphilis patients that Ellery treated with malaria therapy at Mont Park during this period. This methodology avoids some of the sampling risks when dealing with large populations over larger timeframes. Furthermore, I have been able to compare the specific outcome of each individual patient as recorded by Ellery for his published outcomes with that recorded in the medical record archives. This adds to the rigour of my research.

A broader methodological issue to flag is the issue of whether it is valid for historians to make judgements about a historical treatment’s effectiveness, beyond just what the physicians, patients and observers of the era thought about it. This is a complex and debated topic (for different perspectives see Braslow, 1997: 4–5; Sadowsky, 2017: 5–10). Importantly, much of my critique, although sometimes couched in present-day clinical research language, aligns with critiques made by malaria-era physicians who questioned some of the enthusiastic claims made for malaria therapy (Bolton, 1929; Clayton, 2022; Reid, 1932; Stokes, 1934: 1177–1178).^4^ Thus, I would argue, my critique of Ellery’s research and his enthusiastic claims about malaria therapy is not illegitimately anachronistic or presentist.

## Reginald Ellery and the Mont Park malaria fever clinic, 1927–1928

Mont Park Mental Hospital is built on an estate of about 1300 acres splendidly situated . . . it is close enough to town to be accessible by the patient’s relatives and far enough out to possess the advantages of country air and surroundings. The country is undulating with magnificent views of the Dandenong Ranges towards the north-east (Farran-Ridge, 1933: 239).

Mont Park, located on the northern outskirts of Melbourne, was founded and designed by Victoria’s Inspector General of the Insane, William Ernest Jones. It opened in 1913 (Inspector General of the Insane, 1913: 43) and by 1929 was described as being overcrowded, housing around 1200 patients (Inspector General of the Insane, 1930b: 16). In the 1930s and 1940s, Mont Park was considered Victoria’s leading hospital for innovative treatment of mental illness. It was here that the state’s malaria fever therapy clinic operated. In 1937, insulin coma therapy was first introduced into Australia at Mont Park (Cade, 1973). In 1954, the Mont Park surgical unit opened so leucotomies could be performed onsite (previously, select cases had been referred to the general hospitals for this surgery) (Mental Hygiene Authority, 1955). Mont Park closed as an inpatient unit in the 1990s, as part of the era’s sweeping deinstitutionalization policies (Department of Health and Human Services, 2016).

In 1926, Mont’s Park’s “New Hospital” ward opened. This was described as favourably modern, with “conditions indistinguishable from those of a general hospital” (Farran-Ridge, 1933: 239). It was used to accommodate voluntary patients, as well as certified insane patients who were seriously ill or who were undergoing special forms of treatments (Farran-Ridge, 1933), with patients “nursed assiduously by a very competent staff under the best conditions” (Inspector General of the Insane, 1927: 25).

In 1927, junior psychiatrist Reginald Ellery who, along with John Adey, had undertaken the first trials in Victoria of malaria therapy at the Sunbury Hospital for the Insane in 1925–1926 (Clayton, 2022) was transferred to Mont Park to run the newly established Mont Park “malaria fever clinic” at the “New Hospital” (Inspector General of the Insane, 1928: 27; 1930a: 14). The decision to run a specific malaria therapy clinic may have been more due to the influence of the reports of malaria’s success emanating from England than Ellery’s Sunbury trials, the results of which Ernest Jones described as “scanty and unsatisfactory” (Inspector General of the Insane, 1927: 37–38).

Reginald Ellery, despite his youth, already had a chequered career. In 1924, he had been the subject of a Victorian Royal Commission into allegations of cruelty and experimenting on patients and had been only partially exonerated (Kelley, 1924).^5^ As Ellery (1956) bitterly described it, he had subsequently been sent “for his sins” to the Sunbury hospital (p. 149). However, his malaria therapy trials at Sunbury and his published accounts of its success rehabilitated his reputation. This is indicated by his transfer to the more prestigious Mont Park hopsital to oversee the malaria clinic and by being invited, in 1928, to deliver the University of Melbourne’s Beattie Smith Lectures on Insanity on the topic of neurosyphilis and its treatment. In his autobiography, Ellery (1956) recalled that this was “a singular honour to fall a man barely past his thirty-first birthday” (pp. 153–163).

Ellery has been described as “impetuous, provocative and confrontational” (Kaplan, 2014: 572).^6^ Ellery was also enthusiastic and energetic (Inspector General of the Insane, 1928: 27). He even went to the effort to produce a silent film of his practice of malaria therapy at Mont Park to show at his lectures.^7^ The film depicts malaria therapy as a highly successful treatment. It also frames psychiatry as scientific and laboratory based – illustrating Ellery’s desire to help deliver psychiatry from the “prisoned enclosure of the lunatic asylum” and to bring it into the fold of general medicine and neurology (Ellery, 1929a: 74). Ellery later recalled that malaria raised the status of psychiatrists “in the mental hospitals from that of custodians to clinicians . . . Asylum medical officers were . . . invited to medical meetings and listened to with interest. A budding respect became gradually perceptible” (Ellery, 1956: 154). This history indicates that whatever else it did, malaria therapy advanced Ellery’s personal career and, additionally, more broadly, it advanced the status of the whole psychiatric profession.^8^

The Mont Park malaria fever clinic, with Ellery in charge, opened in mid-1927. Any certified insane patients diagnosed with neurosyphilis, including a growing number of voluntary patients, were channelled to Mont Park for malaria therapy (Inspector General of the Insane, 1928: 27, 1930a: 21). In Victoria, there was an effort to undertake blood Wassermann testing on all patients admitted to Victoria’s mental hospital – although the pathologist, William Lind, complained this was not occurring (Inspector General of the Insane, 1928: 30). Most of Ellery’s malaria-treated patients also appear to have had a cerebrospinal fluid (CSF) Wassermann test. Patients were diagnosed with neurosyphilis according to their clinical presentation and Wassermann results. In doubtful cases, Ellery undertook additional testing such as CSF protein, white cell count, and a colloidal gold test (EM/SLV, 1931: 15). Cardiovascular, renal, and respiratory diseases, as well as debility, were described as contraindications for malaria treatment (Ellery, 1929b).

In terms of patient or relative consent, the available sources give no indication that any such consent was obtained from the certified insane patients, however it is likely that voluntary patients gave implicit consent.^9^ This is in keeping with Australian historian, Garton’s (1988) account that during this era “psychiatrists had the right to provide essential treatment for certified patients regardless of consent” (pp. 169–170). Garton noted the fine line between what could be considered “essential treatment” versus experimentation and concluded that some psychiatrists allowed their enthusiasm for potential therapeutic breakthroughs to override concerns of harmful side effects, with patients suffering the consequences. Interestingly, he singled out Ellery’s enthusiastic claims of the success of cardiazol convulsive therapy in the early 1930s, claims that were not substantiated later by other clinicians’ reports (p. 169).

Malaria therapy at Mont Park involved inoculating the patient with “benign tertian” malaria-infected blood, initially obtained from a general hospital patient suffering acute malaria, and subsequently via blood transferred from one infected neurosyphilis patient to the next.^10^ The blood was injected intramuscularly (between the scapulae).^11^ Ellery aimed for the patient to have 12 rigours with temperatures reaching over 104 Fahrenheit. The patients received intensive nursing and medical care and “liver feeding” during the inter-paroxysmal periods, before quinine was used to terminate the malaria illness. There was ongoing attention to a convalescent diet and the patients subsequently received weekly tryparsamide injections for approximately 16 weeks (Ellery, 1929b; EM/SLV, 1931: 46; Inspector General of the Insane, 1928: 39).

The Mont Park malaria fever clinic was not an immediate success. In mid-1927 only a handful of patients were inoculated, and most appeared to have died due to a malignant strain of malaria being used. Discrepancies between the various archival documents mean it is hard to be sure of the exact number of the mid-1927 malaria-treated patients who died of the malignant malaria strain. By comparing the reports in the various documents, it seems at minimum it was four, but it may have been as high as six (Cade, 1979: 32; CID; Inspector General of the Insane, 1927: 39; MPPMR).

In his publications, Ellery largely avoided mention of the deaths of his 1927 malaria therapy patients (Anonymous, 1929; Ellery, 1929b, 1956). On the one occasion he did, he underplayed the number of deaths (Ellery, 1929c). Fear of censure likely contributed to Ellery guardedness about these deaths. No doubt, the ordeal, including the negative press attention, that he endured at the 1924 Royal Commission loomed large.^12^ Once the malignant nature of the mid-1927 malaria strain was recognized, malaria therapy was put on hold at Mont Park until blood infested with a suitable strain of malaria could be obtained (Inspector General of the Insane, 1927: 38).

In the meantime, Ellery reported using tryparsamide as a stand-alone treatment for four GPI patients. Three of whom were described as in the advanced stages of GPI – one described as a bedridden male having numerous seizures. These patients all achieved remissions, with three being discharged, two of whom were employed – which was Ellery’s definition of clinical cure (Ellery, 1928, 1929b; EM/SLV, 1931: 46). Intriguingly, in his unpublished thesis, Ellery also reported using quinine as a stand-alone treatment on four GPI patients and described all as having some level of remission (EM/SLV, 1931: 47; EM/SLV, undated). I will discuss Ellery’s successes with tryparsamide and quinine treatment in more detail shortly.

In November 1927, blood infected with a benign strain of malaria was obtained from New South Wales (NSW) Lunacy Department and was transferred via train in a “thermos on ice” to Mont Park (Ellery, 1929c; EM/SLV, 1931: 69). Ellery’s published account of his November 1927 to December 1928 malaria-treated neurosyphilis patient cohort (*n* = 90) described the outcomes as: 76% (*n* = 69) improved, around half of which were “clinical cures” (which Ellery defined as discharged and return to employment); 42% (*n* = 38) discharged; the remainder 23% (*n* = 21) unimproved; and no deaths from treatment (Table 1) (Anonymous, 1929; EM/SLV, 1929a). Ellery also reported these figures in the Inspector General of the Insane Report for the Year 1928, where he also noted that five of the improved but not discharged patients had subsequently died (Inspector General of the Insane, 1930a: 14–15).

**Table 1.** Outcomes of Mont Park patients diagnosed with neurosyphilis and treated with malaria therapy in 1928 according to Ellery’s published account.^#^

Outcome	Number (total 90)	Percentage
Discharged	38	42.2
Improved	31	34.4
Unimproved	21	23.3
Deaths due to or during treatment	0	0

Source: ^#^As reported by Ellery at the Australian Medical Congress, September 1929 (Anonymous, 1929).

Ellery described that his 90 malaria-treated neurosyphilis patients “had not been selected” and “had been taken in all stages from that of mild euphoria and facial tremor to the advanced condition approaching the classical third stage of general paralysis”, with 85% (23 patients, out of a total 27 patients) early-stage patients being discharged (Anonymous, 1929: 503).

Ellery contrasted these outcomes favourably to a spontaneous remission rate of 4% in untreated GPI patients as reported in the literature. Thus, he claimed, malaria therapy was an effective treatment for GPI (Anonymous, 1929; Ellery, 1929b; Inspector General of the Insane, 1930a: 15). He concluded that:

Malaria therapy is of definite value in all cases of syphilis involving the central nervous system . . . for without some such treatment . . . practically all these people would now be among those demented wretches into the twilight of whose minds ideas have ceased to cast their shadow (Ellery, 1929b: 112–113).

At a superficial glance, Ellery’s published outcome rates seem a dramatic and convincing demonstration of malaria therapy’s effectiveness. However, as I will now demonstrate, a deeper evaluation that considers important changes in diagnostic and institutional practices and scrutinizes the medical record archives is far less convincing.

## Changing diagnosis and nomenclature – GPI becomes neurosyphilis

The first important point to highlight is the shift from the diagnostic category of “general paralysis of the insane” to that of “neurosyphilis”. This is reflected in the titles of Ellery’s articles. The titles of Ellery’s earlier articles indicate he is treating “general paralysis” (Ellery, 1926, 1927); whereas by 1928 the titles of his later articles (and his silent film) indicate he is treating “neurosyphilis” (Anonymous, 1929; Ellery, 1929a, 1929b; NFSA/Ellery, 1925). A diagnostic drift from GPI to neurosyphilis would have significantly impacted on the issue of malaria therapy’s effectiveness, as I will now explain.

The era’s classification, like 21st century classifications, typically distinguished several forms of neurosyphilis. GPI was generally recognized as the most severe, the least responsive to standard arsenical treatments, and having the worse prognosis (Moore, 1933: 340–341, 376–378) (see Table 2).

**Table 2.** Neurosyphilis classification and prognosis.

Type	Timing	Prognosis (if no treatment)	Clinical presentation
ANS (asymptomatic neurosyphilis)	Early (within 4 years of initial infection) or late (after 4 years initial infection)	Mild changes (Grade I) could spontaneously resolve; marked abnormalities (Grade III) indicated likely later progression to GPI	No clinical features but cerebrospinal fluid findings of neurosyphilis (e.g. Wassermann positive, increased white cells and globulin levels, colloidal gold test positive)
Meningeal	Early (months to several years after initial infection)	Variable	Headache, meningitis symptoms, stroke
Meningo-vascular/cerebrospinal	Medium to late (5–12 years)	Recovery likely, but with ongoing deficits and chronic invalidism; Death uncommon	Stroke; neuropsychiatric clinical presentations that could overlap with GPI presentation
Parenchymatous-tabes dorsalis	Late (over 10 years after initial infection)	Protracted course over many years, often with chronic invalidism; sometimes mild with little progression	Argyll-Robertson pupils, abnormal reflexes, impaired sensation, ataxic gait, Charcot’s joints, lightening pains, bladder dysfunction, blindness
General paralysis of the insane	Late (over 10 years after initial infection)	80% patients died within 4 years of onset; complete remission (for weeks to months, occasionally years) in up to 5% and incomplete remissions in up to 15%	Wide range of progressive neuropsychiatric symptoms starting with a prodromal phase (e.g. changed personality, fine lingual tremor) and progressing to frank neuropsychiatric manifestations (e.g. mania, cognitive impairment, depression, gait impairment, slurred speech, Argyll-Robertson pupils)
Gumma of the CNS	Late	Dependent on location	Symptoms of space occupying lesion
Congenital neurosyphilis	Transmitted to the infant during pregnancy and birth		Intellectual impairment and physical stigmata; Development of clinical features of GPI in late teenage or early adult years

Source: Derived from Moore (1933) and Stokes (1926). This classification is similar to present day classifications (Hamill et al., 2024; Daey Ouwens et al., 2019).

But in 1928, Ellery espoused a quite different theoretical viewpoint. Ellery set out what he called, a “unity in variety” concept of neurosyphilis. He wrote: “The neurosyphilitic is usually a neurosyphilitic and not merely a spinal syphilitic or a cerebral syphilitic” (Ellery, 1929a: 74). He believed that the traditional division of neurosyphilis into the meningo-vascular type and parenchymatous syphilis (GPI or tabes dorsalis) type was not in line with contemporary science. Instead, he argued that neurosyphilitic phenomena needed to be viewed as a “unity in variety” (p. 78) wherein any spirochaete invasion of the central nervous system eventually led to GPI:

It is true that in the past the clinical differentiation of neurosyphilis into types has been of considerable prognostic significance – the meningo-vascular variety being more amenable to routine treatment, but with the advent of malarial therapy. . .this differentiation is not of such importance. And my own series of cases, to which I shall presently report, contained examples of practically all clinical types. Believing as I do that any case of neurosyphilis may sooner or later show cortical involvement and the subsequent harm of mental symptoms, I have not hesitated to give malaria to all cases coming to treatment to whom the examination of the cerebrospinal fluid shows undoubted evidence of syphilitic infection (EM/SLV, 1929a: 7–8).

Ellery recommended that the problem of neurosyphilis should be treated “prophylactically” by examining the cerebrospinal fluid of all patients with primary or secondary syphilis and then correcting those abnormalities with “appropriate” treatment before discharging the patient as cured (Ellery, 1929b: 114).^13^ As the above quote indicates, he seems to have considered “appropriate” treatment to be malaria therapy.

Many syphilologists and psychiatrists of the malaria era recognized that there were patients who presented with mixed clinical and pathological features of various types of neurosyphilis. However, Ellery’s “unity in variety” position was never widely adopted, at least according to the English-language literature. Instead, distinctions continued, and continue, to be made between the different varieties based on clinical presentation and response to treatment (Daey Ouwens et al., 2017, 2019; Hamill et al., 2024; Moore, 1933: 333–334) (see Table 2). Furthermore, differentiating meningo-vascular neurosyphilis from GPI could be difficult, and prior to the introduction of malaria therapy and tryparsamide, treatment responsiveness or death could be the defining feature – with patients who appeared to respond to treatment classified as having non-GPI forms of neurosyphilis (Davis, 2012; Minogue, 1926; Stokes, 1926: 928).

The key point to underscore is that Ellery’s Mont Park malaria-treated patients included a mix of GPI and other better prognostic types of neurosyphilis. Thus, we would expect these patients would have comparatively better outcomes when compared to the traditional pre-malaria-era certified-insane GPI patients.

## Early diagnoses and an unreliable Wassermann test: False-positive diagnoses

The second point to note is that the emphasis on early neurosyphilis diagnoses would have increased the risk of false-positive diagnoses. Early diagnosis relied on “non-specific” neurological and psychiatric symptoms, which could also be found in non-neurosyphilis conditions or even “normal” patients (Moore and Hopkins, 1930). For example, Ellery diagnosed patients as having neurosyphilis when only mild clinical features, such as “mild euphoria” or a “facial tremor”, were present (Anonymous, 1929). In such cases Ellery would have relied largely on the findings of the blood and cerebrospinal fluid Wassermann test to make the diagnosis of neurosyphilis. However, the Wassermann test, particularly the “one-tube method” at use in Victoria’s Lunacy Department, was notoriously at risk of false-positive results. This seems to have been something Ellery became more aware of in 1930.

In late 1928, William Lind, the Victorian Lunacy Department’s long serving pathologist, suddenly died. Ellery left his role at the malaria clinic and took over the role of the Lunacy Department’s pathologist. Ellery found a 24% rate of positive blood Wassermann results for patients admitted to Victoria’s hospitals of the insane during 1929. He considered this too high to be correct (Inspector General of the Insane, 1930b: 19). This led him to up-date the Wassermann technique:

A necessary modification of the Wassermann test was inaugurated this year, relinquishing the old one-tube method for a four-tube method, similar to that in use at the Baker Medical Research Institute at the Alfred Hospital, which makes for greater accuracy (Inspector General of the Insane, 1931: 20).

This updated method of Wassermann testing had been recommended for over a decade because of widespread international concerns regarding the accuracy of the “one-tube” method (Fairley and Fowler, 1921).^14^ Ellery reported that, using this new method, the percentage of admissions in 1930 with a positive blood Wassermann was 15.6% (Inspector General of the Insane, 1931: 20). This was a drop of over 8% from the preceding year. Nevertheless, Ellery does not appear to have reflected on the possible implications of false-positive Wassermann results on his 1925–1928 malaria therapy trials at Sunbury and Mont Park. However, it seems reasonable for us to infer, given the problems Ellery identified with the Wassermann test technique, that it is likely that some of his malaria-treated patients may not have had neurosyphilis. This raises further doubts about the validity of Ellery’s reported outcomes at both institutions.

## Voluntary neurosyphilis patients at Mont Park

The third important point to highlight is that many of the new cohort of patients diagnosed with early neurosyphilis were admitted to Mont Park as “voluntary boarders”. In other international contexts it has been reported that voluntary admissions of neurosyphilis patients to public mental hospitals prior to malaria therapy were virtually unknown (Braslow, 1997: 85). Mont Park’s medical records indicate a similar situation in Victoria. The first year that Victorian legislation allowed voluntary boarders to be admitted to Victoria’s hospitals for the insane was 1915 (Inspector General of the Insane, 1915: 59; Victorian Statutes, 1915). From 1915 until 1926 there were only a small number of voluntary boarders – between one and eleven – admitted to Mont Park each year. Only one such patient was listed as having a syphilis-related diagnosis (MPVBR). But in 1928, there was a marked increase in voluntary boarders, with 72 being admitted. Of these, 32 (44%), had a syphilis-related admission diagnosis (MPVBR) and were admitted to the New Hospital to be given malaria therapy (EM/SLV, 1929b; Inspector General of the Insane, 1930a: 21; MPVBR). Ellery indicated that many of these voluntary boarders had been admitted from the Melbourne Hospital (now the Royal Melbourne Hospital) where he had a part-time position as clinical assistant to the hospital’s psychiatrist (Ellery, 1956: 154, 158).

Ellery described the voluntary patients as being a different “class” than involuntary patients (Ellery, 1956: 158). Voluntary patients paid for their “maintenance” at the hospital (Victorian Statutes, 1915: 3011–3012). I have found no evidence, but we can speculate that this financial incentive might have contributed to some enthusiasm for the cash-strapped Victorian Lunacy Department to set up the malaria therapy clinic. Furthermore, increasing numbers of “higher-class” patients being admitted and treated with malaria would have almost certainly contributed to malaria therapy’s apparent effectiveness. This is because socioeconomic grouping tends to be associated with factors such as general health status, nutritional status, family support, and early access to medical treatment. Such factors are likely to impact positively on treatment outcomes.

Notably, only 41% of Ellery’s voluntary malaria patients were diagnosed with GPI, the remainder were given a variety of diagnoses (MPVBR). These diagnoses included “locomotor ataxia” (another term for tabes dorsalis), melancholia caused by syphilis, hallucinations caused by syphilis, hypochondriasis caused by syphilis, drug addiction, obsessional ideas and worry. Some had no diagnosis listed at all. In contrast, 84% of Ellery’s malaria-treated involuntary patients had a recorded diagnosis of GPI (MPADR). This difference in diagnoses suggests that the voluntary neurosyphilis patients would have been clinically quite different from the historical involuntary GPI patients to whom Ellery compared them when considering outcomes. This is also suggested by Ellery’s film, which shows quiet and cooperative patients, seemingly very different from the descriptions of the certified insane GPI patients that Ellery had treated at Sunbury only 2 years earlier (see Clayton, 2022).

Table 3 provides a summary of the outcomes of Ellery’s voluntary malaria-treated neurosyphilis patients as recorded in the MPVBR. The reader can observe that the voluntary patient cohort had a much higher (86%) discharge rate, compared to the discharge rate (42%) of voluntary and involuntary patients combined (see Table 1).

**Table 3.** Outcomes of Mont Park voluntary neurosyphilis patients treated with malaria in 1928 according to the medical records^#^.

Outcome	Number (*n* = 29)	%	Comments
Discharge before July 1929	25	86	
Death before July 1929	2	7	
Death as inpatient after July 1929	1	3	transferred to involuntary status and died as an inpatient in 1962
Discharge after July 1929	1	3	

Source: ^#^Outcome as indicated by medical records (derived from data in the MPVBR).

The medical records also shows that the voluntary patient cohort had a much higher (86%) discharge rate when comparted to the involuntary patient cohort alone (22%) (Table 4).

**Table 4.** Outcomes of Mont Park involuntary neurosyphilis patients treated with malaria in 1928 according to the medical records^#^.

Outcome	Number (*n* = 59)	%	Comments
Discharge before July 1929	13	22	
Discharge July 1929–December 1933	7	12	
Discharge after 1933	3	5	One patient was discharged in 1939 recovered; Two were discharged in the 1950s as unimproved
Death before July 1929	14	24	Four of these patients died within 6 weeks of malaria inoculation.
Death July 1929–December 1933	12	20	
Death after 1933 (inpatient)	7	12	This death rate maybe higher as this figure does not include the patients transferred to the regional hospitals
Transferred to a regional SHI	3	5	Final outcomes unknown.

Source: ^#^Outcomes derived from data in MPADR, MPPMR, and CID.

In sum, Ellery’s comparisons of the outcomes of malaria-treated patients, which included the voluntary patients as well as involuntary patients, to non-malaria treated involuntary patients only, would almost certainly have favoured malaria due to clinical and demographic differences between the cohorts. This is borne out by a comparison of Ellery’s voluntary versus involuntary cohort discharge rates, which shows a four-fold higher discharge rate for the former.

## Adjunct interventions

Fourthly, it is important to highlight that malaria therapy involved a lot more than just inoculation of the patient with the malaria parasite. As I have described above, malaria therapy involved attentive nursing and medical care in the modern hospital ward, nutritional supplemental food (including “liver-feeding”),^15^ quinine to terminate the malaria illness and an intensive post-malaria course of tryparsamide. And this treatment package came with a healthy dose of hopeful expectation.^16^ All these additional features could have exerted a therapeutic effect independent to any specific curative biological effects of malaria infection, which was the mechanism of action posited in the era (Wagner-Jauregg, 1936).

For example, tryparsamide was a novel arsenical compound developed by researchers at New York’s Rockefeller Institute in 1915 and first trialled as a treatment for syphilis in the early 1920s (Davis, 2008: 173). It reportedly had more efficacy for GPI than the preexisting arsenical medications, which was the reason why Ellery chose it as an adjunct for malaria therapy (EM/SLV, 1931: 45). American syphilologist, John Stokes, summarized what was known about tryparsamide’s effectiveness when used alone for GPI. He reported that it achieved complete remission in approximately one third of patients, partial remission in a second third, and no benefit in the remaining third. Stokes (1934) suggested that the results of malaria therapy plus tryparsamide versus tryparsamide alone were similar (p. 1191). From 1927 onwards, Ellery routinely combined malaria and tryparsamide treatment for his neurosyphilis patients (Inspector General of the Insane, 1928: 39). However, as previously mentioned, he also reported using tryparsamide as a stand-alone treatment in four GPI patients, all of whom reportedly achieved remission.

Another intriguing factor we should consider when evaluating malaria therapy’s effectiveness is quinine, which was used to terminate the malaria illness. As mentioned above, Ellery seems to have considered the possibility that quinine might have had its own therapeutic impact. He reported that he trialled quinine alone on four GPI patients:

Experiments were conducted into the effect of quinine on four untreated paretic patients when the drug was “pushed” to the limits of tolerance. Each case showed a remission of symptoms to a varying degree which lasted from two to six months (EM/SLV, 1931: 47).

Ellery described that one of his quinine-treated patients, an exalted and very noisy “paretic” became an “industrious ward-worker and a rational talker”.This patient was subsequently given malaria (no information is provided on whether malaria led to any further clinical improvement) and remained well for over 2 years (p. 47). Ellery concluded that quinine acted as a general tonic, much like tryparsamide and was an important accessory to malaria.

## Published versus medical records’ accounts

Fifthly, my research finds that the medical records’ account is far less positive than Ellery’s published accounts of malaria therapy at Mont Park. I have already described that the medical records show that Ellery’s involuntary malaria-treated patient cohort – which would have been more like the pre-malaria-era GPI cohorts than the voluntary cohort – had a markedly lower discharge rate (22%) than the whole (voluntary and involuntary patients combined) cohort (42%) (see Tables 2 and 4). Ellery only published this latter figure.

Furthermore, there were more deaths after malaria treatment, including shortly after malaria inoculation, than Ellery’s published accounts acknowledged. In his published accounts, Ellery claimed there had been no deaths resulting from malaria treatment in the 1928 cohort of patients (Anonymous, 1929). In the Inspector General of the Insane (1930a: 14–15), Ellery also claimed that there had been no deaths as the result of malaria therapy but that five patients, who experienced benefit from malaria but had not been able to be discharged, had subsequently died. However, the Mont Park records paint a far more negative picture. They indicate that 14 of Ellery’s involuntary 1928 malaria-treated patients died between the start of 1928 through to June 1929 (see Table 5). Of these, four patients died less than 6 weeks after malaria inoculation. In addition, the Mont Park records (MPPMR) indicate that another involuntary patient, not included in Ellery’s list (EM/SLV, 1929b), died 2 weeks after malaria inoculation. Moreover, one of the patients that Ellery counted as a discharge, in fact had died – I discuss this patient in more detail shortly. Also, notably, Ellery wrote the medical reports for most of the coroner inquest depositions, which were mandated by legislation for any certified insane patient who died. On multiple occasions, including for those deaths that followed shortly after malaria inoculation, he failed to disclose that the patient had received malaria therapy. Ellery, either made no mention of the treatment or vaguely alluded to “special treatment” (CID).

**Table 5.** Deaths of Mont Park patients with GPI/neurosyphilis January 1928–June 1929 according to the medical records^#^.

Patient sex (M = male, F = female)/age	Admission	Malaria therapy/date	Death as a Mont Park inpatient
1. M/60	9 December 1925	No	24 January 1928
2. M/62	31 October 1927	No	4 February 1928
3. M/38^###^	28 February 1928	Yes/29 February 1928^##^	13 March 1928, not included in Ellery’s List; Ellery’s CID report does not mention malaria
4. M/55	26 July 1927	Yes/12 December 1927	25 April 1928, Ellery’s CID report does not mention malaria. Classified as improved by Ellery
5. F/37	28 February 1928	Yes/11 March 1928^##^	26 April 1928, no mention of malaria treatment in Ellery’s CID report
6. M/59	9 December 1925	No	24 July 1928
7. M/41	5 October 1927	Yes/22 December 1927	27 July 1928, malaria mentioned in Ellery’s CID report
8. M/54	4 May 1928	No	8 August 1928
9. M/38	20 April 1928	Yes/29 April 1928	4 August 1928 Malaria mentioned in Ellery’s CID report
10. M/52	19 June 1928	Yes/27 June 1928	28 September 1928, malaria mentioned in Ellery’s CID report. Classified as improved in Ellery’s list
11. M/46	10 September 1928 (transferred from Kew)	Yes/11 September 1928^##^	20 September 1928, no mention of malaria in Ellery’s CID report
12. M/53	10 July 1928 (transferred from Kew)	Yes/9 August 1928^##^	10 September 1928, malaria mentioned in Ellery’s CID report
13. M/35	10 September 1928 (transferred from Kew)	No	22 September 1928
14. F/52	29 November 1927	Yes/9 June 1928	3 September 1928, CID notes that received malaria
15. M/45	16 April 1928	Yes/20 April 1928	3 December 1928, Ellery’s CID report does not mention malaria. Classified as improved in Ellery’s list
16. F/53	30 November 1926	Yes/9 April 1928	31 December 1928, no mention malaria in CID report/Ellery’s list classifies as improved
17. M/50	5 December 1928 (transferred from Sunbury)	Yes/10 December 1928^##^	15 January 1929, malaria noted in CID report
18. M/47	10 July 1928 (transferred from Kew)	Yes/16 July 1928	20 January 1929, Ellery’s list classifies as a discharged patient/malaria mentioned in CID report
19. M/39	27 September 1927	Yes/22 December 1927	22 June 1929, no mention of malaria in CID report
20. M/48	10 January 1928	Yes/7 April 1928	25 June 1929, no mention of malaria in CID report

Source: ^#^MPPMR and CID.

Note: ^##^Indicates patients who died within 6 weeks of being inoculated with malaria.

### Ellery’s list omitted mention of patient no. 3.

Additionally, Ellery’s inclusion of some patients in the “improved but not discharged” category is questionable as some of these patients died within months of malaria inoculation (patients 4, 10, 15 and 16 in Table 5). The medical records I have reviewed also indicate that one discharged patients’ clinical condition had already improved significantly even prior to receiving malaria, which raises doubt about malaria therapy being the agent of improvement. . Anothers’ discharged patient’s neurosyphilis diagnosis seems dubious, according to the standards of the era, as his CSF Wassermann testing was reported as negative.

Next, despite the “discharge” category being used to signify substantial benefit from treatment, some discharged patients were not clinically improved.^17^ For example, one patient was inoculated with malaria in November 1928. He was sent on trial leave in late December but then continued to return for weekly tryparsamide injections. The last file notes, in June 1929, which was the official discharge date, state that this patient was described by his wife as almost blind, mute, and virtually chair bound. Thus, we can observe that despite discharge, this patient was far from improved; rather he appears to have declined into end-stage GPI.

Of critical importance, is that several patients that Ellery’s List classified as discharged (prior to July 1929) were not in fact discharged. In fact, one was dead due to Ellery accidentally injecting him with morphine instead of tryparsamide (patient number 18 in Table 5). Another is listed in the records as having never being discharged and he died as a Mont Park inpatient in 1940 (MPADR, CID). And yet another is a patient who failed trial leave and remained an inpatient to be given an additional course of tryparsamide in late 1929, with little further benefit. This patient, a WWI veteran, was eventually discharged in 1930, described as a “neurasthenic wrapped up in his own state of health”.

Of additional note is that 10 of the 61 (16%) involuntary neurosyphilis patients that Ellery treated with malaria in 1928 were women (EM/SLV, 1929b). In comparison, considering all GPI involuntary admissions to Victoria’s hospitals for the insane between 1920 and 1926, only 8% were women (Inspector General of the Insane, 1927: 39). This is consistent with other reports of a gradually increasing proportion of females being diagnosed with neurosyphilis in the malaria era compared to the pre-malaria era (Davis, 2008; Hare, 1959). This change may have impacted on outcome results, because neurosyphilis diagnosed in females was reportedly more likely to be a non-GPI form and to have a better prognosis than neurosyphilis diagnosed in men (Moore, 1933: 217–218).

And, finally, counter to his claims, Ellery’s malaria-treated patient group was unrepresentative because of the exclusion of certain patients, such as those who had rapidly progressing fatal GPI, those who had debility from advanced GPI, and/or those who had serious comorbid medical conditions. The medical records indicate there were five such involuntary neurosyphilis patients at Mont Park (patients 1, 2, 6, 8 and 13 in Table 5) and a further four at Royal Park Receiving House who died in 1928 and were not given malaria (Ellery/SLV; CID; MPPMR). Thus, some of the worse prognostic neurosyphilis patients were not selected for malaria therapy. This is likely to have been a clinically reasonable decision. However, it would have the effect of exaggerating malaria therapy’s positive outcome results when the malaria-treated patients were compared with pre-malaria-era cohorts that included these types of patients, or with the outcomes of contemporaneous cohorts not given malaria.

In sum, the medical record account indicates a far less rosy picture of malaria therapy’s therapeutic impact than that claimed by Ellery in his published accounts.

## Mont Park malaria clinic 1929–1950

Ellery departed the Mont Park malaria clinic in 1929, but the clinic continued under the leadership of other psychiatrists.^18^ My review of various archival published documents indicates that the success rates of malaria therapy as reported by Ellery were not maintained in the subsequent years of its use.

For example, Prendergast (1939), a psychiatrist at Mont Park, reported on malaria therapy for patients diagnosed with GPI. He described that 66 male voluntary and involuntary GPI patients had been admitted to Mont Park between 1936 and 1938 and given malaria therapy in combination with tryparsamide. He reported a 13.5% remission rate. These were patients who he described as being able to leave hospital and have some type of employment, often at a lower level than previously and “with only the exceptional patient able to resume anything like a responsible position” (p. 363). A further 21% of patients were able to be discharged but were not employable. Of the rest, 36% experienced no change, 16.5% worsened, and there was a 12% death rate. Despite these rather dismal figures, Prendergast remained a believer in malaria therapy, arguing that these poor results were due to GPI patients presenting too late, with many having more than a 6-month clinical history prior to admission. However, this does not seem to adequately explain the differences in his outcomes compared to those reported a decade earlier by Ellery, as many of Ellery’s patients had an extended duration of pre-existing illness. Another example of more pessimistic malaria outcome results is the Director of Mental Hygiene Report (1947: 21), which described that 29 neurosyphilis patients were given malaria, resulting in three (10%) recoveries and two (7%) “social” remissions. Seven (24%) of the patients died.

We need to be mindful that, along with methodological limitations and differences between the studies, other complex contextual elements may have changed over the period and could also have impacted on GPI outcomes. This muddies our attempts to interpret the reported changes in malaria therapy’s effectiveness over time. However, my view is that, in Victoria, the available evidence indicates that malaria therapy did not live up to its initial promise, certainly not as promulgated by Ellery, as a cure for GPI. This may be most starkly illustrated by noting the contrast between the outcomes of malaria therapy as reported by Ellery (1926, 1927) and Prendergast (1939). Ellery (1926, 1927) reported that malaria therapy (not combined with tryparsamide) led to some level of remissions in 70% involuntary male GPI patients, with 30% being “apparently cured”. Whereas Prendergast (1939) noted that malaria and combined tryparsamide treatment led to only a 13.5% remission and return to employment rate (Ellery’s definition of “clinical cure”) in male GPI patients (a mix of voluntary and involuntary) with only the exceptional patient returning to a responsible work position.

## Malaria therapy in other Australian states

Following Ellery’s published reports, malaria therapy was soon being implemented in other Australian mental hospitals – with infected blood being transferred in thermos flasks “on ice” from NSW and Victoria to other states (Hogg, 1929). At the September 1929 Australian Medical Congress, several psychiatrists other than Ellery reported on their experiences with malaria therapy (Anonymous, 1929). The most significant report, aside from Ellery’s, was Clifford Henry’s from Sydney’s Callan Park mental hospital. Over the preceding 2 years he reported giving malaria to 190 neurosyphilis patients. He compared their results with 349 patients admitted with GPI in the pre-malaria-era years. Thus, this was a comparison comparing historical GPI patients to malaria-treated neurosyphilis patients, with all the limitations I have previously described. Henry described the outcomes as: 15% of the malaria-treated neurosyphilis patients were discharged (with half earning a living), this compared to 6.6% of pre-malaria-era GPI patients able to be discharged (none earning a living). A further 19% of the malaria-treated patients were greatly improved 2 months following malaria, in comparison only 2% of pre-malaria-era patients were greatly improved 2 months after admission. Two months after admission, 11% of the malaria-treated patients were dead, whereas 21.5% of the non-malaria group were dead. Thus, whilst both Ellery and Henry give us statistics in favour of malaria therapy, there appear to be some notable differences between their findings. For example, Henry’s reported 15 % discharge rate for malaria-treated patients is much less than Ellery’s 42% rate. Similarly, his 7% “earning a living” rate is markedly less than Ellery’s 30%–35% figure. The report of the Congress discussion indicated that some psychiatrists expressed concern regarding deaths caused by malaria and noted that good results could be obtained from tryparsamide alone; others disputed the conflating of all neurosyphilis with GPI and noted the doubts this raised about the reliability of the reports of malaria’s benefits for GPI. However, despite these misgivings, overall, those present at the Congress seemed to agree that good results could be obtained from malaria therapy in GPI, and that combined malaria and tryparsamide treatment was to be commended.

Subsequently, between 1929 and 1935 there are scattered articles and case reports in the *Medical Journal of Australia* regarding the treatment of neurosyphilis. Some patients were reported as being treated with malaria therapy and others with tryparsamide alone. It is also notable that several of the case reports indicate neurosyphilis patients had physical and mental symptoms, intermittent in nature, of many years’ duration prior to their current hospital admission and malaria or tryparsamide treatment. This speaks to the difficulties of attributing improvements in neurosyphilis symptoms to the impact of malaria. The most significant article, beyond those already mentioned, was a progress report on the routine practice of malaria therapy at Callan Park, by Henry (1932). He reported that 26 % of neurosyphilis patients treated by malaria (usually with adjunct tryparsamide treatment) were able to be discharged, but only 7% were able to earn a living. Again, it needs to be noted how these figures, like his 1929 report, are markedly less positive than Ellery’s claims of a 30%–35% “clinical cure” (patients discharged and able to work).

## Penicillin and the demise of the Mont Park malaria clinic

Internationally, penicillin was first used to treat infections in early 1943 (Magner, 2009: 63–64). By October 1943 it was being tried at America’s Johns Hopkins hospital, for patients with neurosyphilis, either alone or in combination with malaria therapy (Reynolds et al., 1946). At Mont Park, penicillin appears to have been first used for neurosyphilis in 1945 (Director of Mental Hygiene Report, 1946: 23). My review of the reports, up until the year 1955, finds that 1947 is the last year that malaria therapy is mentioned.

What about GPI? In the Reports up until 1952, GPI continued to be noted as a diagnosis and cause of death. In 1952 it was recorded as a diagnosis in 12 patients, two patients were described as recovering and eight patients died (Mental Hygiene Authority, 1953: 14). In the 1954 Report, GPI was no longer listed as a diagnosis in the statistical forms. That year the superintendent of Royal Park Receiving House wrote that “general paralysis of the insane has virtually disappeared” (Mental Hygiene Authority, 1954: 31). The introduction of penicillin seems an obvious explanation for this. However, other factors might have been involved as British psychiatrist, Hare (1959) documented a declining incidence and severity of GPI that had commenced prior to both malaria therapy and penicillin. (Hare’s thesis was that an evolutionary change in the syphilis spirochete, with resulting changes in its neurotoxicity, might have accounted for this.) Interestingly, a similar declining rate of mental hospital GPI admissions dating back to 1920 had also been noted in Victoria Inspector General of the Insane, 1927: 37; Stoller and Emmerson, 1969).

## Conclusions

The Mont Park malaria fever clinic opened in 1927 with a zealous Reginald Ellery at its helm. Ellery left the clinic in 1929, but malaria as a treatment for neurosyphilis continued at Mont Park until around 1950. By this time neurosyphilis/GPI admissions were dwindling to become virtually non-existent, and penicillin had been introduced as a treatment for all forms of syphilis.

My study of malaria therapy at Mont Park provides a markedly less optimistic picture of its therapeutic impact than claimed for it by Ellery. I have demonstrated that Ellery’s claims of malaria’s effectiveness were based on what we can consider to be unfair comparisons made between the outcomes of malaria-treated neurosyphilis patients and the outcomes of historical GPI patients. These cohorts had notable differences. Firstly, patients were selected for malaria therapy, with patients being excluded because of neurosyphilis severity and/or general debility or comorbidities – such patients were likely to have the worse prognostic outlook. Ellery’s malaria group also received tryparsamide (and quinine) as an adjunct therapy, neither of which were given to historical GPI patients. Tryparsamide was widely used as a stand-alone treatment for GPI, including by Ellery, with reportedly good effect. Ellery also reported some benefit from use of quinine alone. Moreover, Ellery’s malaria-treated patients benefitted from better standards of nutrition and medical and nursing care, as well as more hope, than non-malaria treated patients. The malaria group were likely to have had better overall health and a higher socioeconomic standing due to the inclusion of voluntary patients. In addition, changing diagnostic practices, with an emphasis on early diagnosis and Ellery’s inclusive “unity in variety” position, surely meant that many of his malaria -treated patients had markedly less severe and less advanced neurosyphilis than the more restricted historical GPI involuntary patient cohorts. All these types of differences would have almost certainly biased Ellery’s findings in the direction of favouring malaria therapy.

Am I only able to make these observations based on my 21st century methodologically informed hindsight? I do not think so. As already discussed, in the 1920s and 1930s there was broad awareness of these types of clinical research issues. Indeed, some physicians in the malaria era were critical of some of the lavish claims made about malaria therapy. They may not have used our current-day research terminology, but they pointed out many of the same issues. To give one example, British psychiatric medical officer, Benjamin Reid, in his 1932 MD Thesis at Glasgow University, aimed to provide a “fairer and clearer” view of malaria therapy and to assess its real value. He wrote:

It has been agreed for some time now that Wagner-Jauregg’s method of treatment does prove beneficial, but much of the work reported has been handicapped by too close a review, too near a vision. The problem under consideration is complicated by the admitted fact that spontaneous remissions of the principal symptoms are known to occur so that some improvement may be expected under practically all forms of treatment. Nothing less than the statistics of a large number of patients in observations extending over periods of time that are measured by years rather than months will justify anything more than tentative conclusions. (Reid, 1932: 1)

From a 21st century perspective, we can observe that even Reid’s own research did not exclude all potential biases and confounding. For example, Reid’s malaria-treated patients also usually received tryparsamide. However, his research, which was contemporaneous with Ellery’s, was much more rigorous and much more measured in its conclusions. Reid reported that only 12% of patients treated with malaria (usually with adjunct tryparsamide) were alive and mentally well 6–9 years after treatment, and while he still thought malaria therapy of value, he described that these results “must diminish a too optimistic outlook” (p. 171). Reid also clearly took the critiques of malaria therapy research seriously, whereas Ellery seems never to have acknowledged the validity of any such critiques; rather he showed a tendency to denigrate those sceptical of malaria (EM/SLV, 1929a). But still, to be fair we need to highlight that Ellery was certainly not alone in his triumphant conclusions about malaria therapy.

Inappropriate comparisons aside, my review of the relevant medical records only adds to the decidedly less positive picture of malaria therapy’s impact on GPI. When the relevant records are compared to Ellery’s glowing published reports, they reveal discrepancies so significant and so consistent they call into question the integrity of his research methods and reporting. Ellery’s published accounts understated adverse outcomes, and he appeared to repeatedly hide deaths. His published accounts also overplayed benefits by misrepresenting cure and improvement. The influence of Ellery’s guiding hand is further borne out by a comparison of the clinic’s initial published results with those of its later years. When Ellery ceased overseeing this work, the apparent effectiveness of malaria fell off sharply, well short of the cure for GPI he touted for it.

In sum, overall, my research findings in this local context do not lend support to claims of malaria therapy being an effective treatment, rather they suggest that a wide range of clinical and research factors could have plausibly created a therapeutic illusion of effectiveness. However, the reader should note these conclusions are, as a historical study of a historical treatment demands, cautious and I make no definitive claims that malaria therapy was ineffective. This current study’s findings echo and expand upon my findings of Ellery’s practice of malaria therapy at Sunbury, 1925–1926 (Clayton, 2022). My findings also largely parallel Davis’s (2008) findings in her study of malaria therapy in the Scottish setting. Further historical research, drawing on the medical record archives, in other localities would further advance our understanding of malaria therapy – such a study of Wagner-Jauregg’s clinic, if records are available, would be of particular interest.

On a final note, it is some interest to read the Director of Mental Hygiene Report (1947). John Catarinich reported that some Mont Park patients had undergone frontal leucotomies:

As regards the results of these operations, I am still of the opinion that a conservative attitude must be adopted. In this, as in most advances in medicine and surgery, there is an initial chorus of rejoicing because of the advances made and claims put forward, which in the course of time, need a good deal of modification . . . in regard to this operation . . . only a small percentage of our patients is likely to benefit (Director of Mental Hygiene Report, 1947: 35).

Catarinich had been Superintendent of Mont Park at the time of Ellery’s introduction of malaria therapy into Victoria. It seems possible that as well as looking forward to lobotomy’s future, he was looking backwards and reflecting on the promise of malaria therapy compared to its eventual results. Catarinich’s words also illustrate that cautious psychiatrists may be Cassandra-like prophets unable to prevent the enthusiastic embracing of new and prematurely celebrated treatments.

## Notes

1. Malaria-era physicians, such as Wagner-Jauregg and Ellery, believed that malaria therapy substantially improved GPI patients’ outcomes compared to no treatment, describing that many more patients evidenced complete or partial remissions – as shown by general symptomatic improvement, decreased mortality rates, increased discharge rates, and increased ability to return to work – and they thought this clinical improvement was a result of a specific biological impact of malaria, albeit the exact mechanism remained unknown (Ellery, 1929b; Wagner-Jauregg, 1927, 1936). This is what I mean in this paper by the term “effective treatment”. This is broadly consistent with present-day understandings of therapeutic effectiveness – an intervention is effective if it has an intrinsic property underlying its causal power to impact on a particular disease/symptom, and not just that a patient’s health improves after an intervention (Ashcroft, 2002; Stegenga, 2018: 40, 112). Present-day medicine sometimes distinguishes between treatment “efficacy” (“a causal property of an intervention which manifests in a particular controlled setting”) and “effectiveness” (“a causal property of an intervention that has the capacity to manifest in settings more general and less controlled that the particular experimental setting in which efficacy was demonstrated”) (Stegenga, 2018: 44). However, in the malaria era this differentiation did not exist and so I do not make this distinction in this paper.

2. Ellery’s List does not clearly indicate the date up until which he calculated outcomes. However, it classifies patients who were discharged according to the medical records as late as June 1929, so this is the date I have used.

3. This information was sourced from the admission and discharge registers, the coroner inquest depositions, and the post-mortem register (CID; MPADR; MPPMR; MPVBR). These sources contain important and detailed information on patient demographics, clinical features, and outcomes. The voluntary patient case notes have not been archived. The involuntary patient clinical notes (PCN) from this era remain unsorted and not generally available but I was able to access those of 10 (16%) of Ellery’s malaria-treated patients. The annual Inspector General of the Insane (later the “Director of Mental Hygiene” and “Mental Hygiene Authority”) Reports to Parliament were another valuable source. These reports were mandated by Victoria’s Lunacy Legislation.

4. It is also important to note, that it has long been recognized that some popular treatments widely claimed as effective may be mere therapeutic illusions. Claude Bernard in 1865 noted therapeutic bleeding for pneumonia, long believed to be efficacious was “a mere therapeutic illusion” and he highlighted the important research principle that “to be valid, comparative experiments have to be made at the same time and on as comparable patients as possible” (Bernard, 1961: 223–224). In regard to how historical physicians judged an intervention’s effectiveness, historiographies of medical research have noted that early in the 20th century although today’s “gold-standard” – the double blind randomized control trial – had not been developed, the wide community of clinical researchers had begun to realize the importance of having concurrent controls and systematic methods for ensuring the equivalence of the treatment and control groups, rather than just case series (Chalmers et al., 2012; Kaptchuk, 1998: 422–424). Notably, Wagner-Jauregg used a random allocation method and control groups, as early as 1900 in his tuberculin trials for GPI, with patients being assigned to treatment or control group according to admission order (Gartlehner and Stepper, 2012). But Wagner-Jauregg, inexplicably, did not use this method for his 1917–1921 malaria therapy trials, which were just uncontrolled case series, as were those of Ellery’s. In 1925 and 1931, Wagner-Jauregg returned to using the random allocation and comparative group method (Gartlehner and Stepper, 2012), but these were not trials designed to test the efficacy of malaria therapy alone compared to no treatment or arsenical treatments alone.

5. This affair received much attention in the press (Anonymous, 1924a, 1924b, 1924c, 1924d, 1924e). The Commission’s findings that largely exonerated the Department and partially exonerated Ellery led to some accusations of it being a whitewash (Anonymous, 1924d: 2). Interestingly, Wagner-Jauregg, just prior to publishing his first accounts of malaria’s success, was also the subject of an official inquiry into allegation against him of cruelty to patients – he was officially cleared, but a later analysis suggests we should hold considerable doubt about the integrity of that inquiry (Eissler, 1986: 143–164).

6. These personality characteristics seem to have created friction with his colleagues. In a 1973 paper, eminent Australian psychiatrist John Cade, alluded to this when discussing the introduction of insulin coma therapy at Mont Park in 1937: “another compelling reason for haste was that Ellery, who had quickly established a tremendous reputation in private psychiatric practice, was returning any day from an overseas tour, in the course of which he had visited Sakel. Naturally it was the ambition of all right-thinking psychiatrists to steal Ellery’s thunder” (Cade, 1973: 1058).

7. Australia’s National Film and Sound Archives holds this film “Incidents in the Treatment of Neurosyphilis by Induced Malaria at Mont Park” (NFSA/Ellery, 1925). This film is archived as circa 1925. However, it seems impossible that the movie was made at this early date. Mont Park’s New Hospital, the setting for many of the film’s scenes, was only opened in 1926 (Inspector General of the Insane, 1927). Ellery, himself, was only transferred from Sunbury to Mont Park in 1927, with successful malaria treatment at Mont Park only commencing in late 1927. Thus, it is most likely that 1928 was the year the film was made. More discussion of Ellery’s production of this film can be found in Kaplan (2013).

8. This is consistent with Davis’s (2008) observation that that the Wassermann test and malaria therapy were central to the psychiatric profession’s claims to be part of modern medicine – scientific and laboratory based (pp. 240–241).

9. Contrastingly, Danish Medical Historian, Jesper Vaczy Kragh, has reported that the introduction of malaria therapy into Danish psychiatric institution in the 1920s led to the first informed consent regulations for psychiatric patients in that country. The Danish health authorities recognized the mortality risk associated with malaria treatment and initiated mandatory informed consent guidelines. Signed written consent were required from either patient or relative prior to the use of malaria therapy (Kragh, 2010). This type of formal legislative response to malaria therapy seems to be an outlier.

10. A problem encountered with this patient-to-patient inoculation method (the alternative method was direct inoculation via infected mosquitos – which had its own problems, as mosquito “farming” was quite complex and risked release of malaria-infected mosquitos into the local area) was that the malaria parasites would only survive in the blood outside the human body for 2–3 days. Ellery noted that the patient-to-patient inoculation method in localities, like Victoria, where malaria was not prevalent meant the malaria strain was at risk of dying out (EM/SLV, 1931: 13, 43, 51). This had happened at Sunbury. Ellery was proud to report that at Mont Park, the strain obtained from NSW in late 1927 was still being used at least 2 years later (Ellery, 1929c). It is possible that the increasing numbers of neurosyphilis patients being admitted for treatment at Mont Park facilitated this strain’s longevity. However, there is another possibility. Ellery wrote that “in mental hospitals where the malarial strain is maintained by inoculating patient to patient, even the most demented patient from whom little or no improvement is expected, may be used” (EM/SLV, 1931: 13). These words hint that Ellery might have been inoculating some patients with malaria purely for the purposes of keeping the malaria strain alive, rather than for the purposes of any expected therapeutic benefit (if so, it seems unlikely that he included these types of patients in his outcome figures). Such ethically troubling practices have recently been identified as having occurred at malaria therapy centres in Austria and the US (Anonymous, 2015; Gambino, 2015; Jahn, 2014).

11. Wagner-Jauregg (1922) reported injecting the malaria-infected blood subcutaneously, while others injected it intravenously (Driver et al., 1926). Sydney psychiatrist, Clifford Henry reported that in contrast to the intravenous method, the intramuscular or subcutaneous method did not need any blood typing (Henry, 1932). However, American psychiatrists reported that they did not ascertain blood group even when using the intravenous method and described the only adverse reaction being a slight rise in temperature in the first 24 hours (Crawford, 1940; Driver et al., 1926).

12. There is an interesting parallel with Wagner-Jauregg who was similarly circumspect, at least in his English-language publications, regarding four GPI patients who died due to a malignant strain of malaria in 1918 (Austin et al., 1992). Wagner-Jauregg did in later publications acknowledge the 1918 malaria therapy deaths, for example in a paper written in 1935 and published after his death in 1946 (Wagner-Jauregg and Bruetsch, 1946).

13. The significance of a positive cerebrospinal fluid Wassermann test in the absence of clinical features of neurosyphilis (called “asymptomatic neurosyphilis” – ANS) was more complicated than Ellery outlined. ANS did not always progress to clinical neurosyphilis. It could spontaneously resolve. It could also resolve with standard arsenical treatment (Moore, 1933: 217, 338–339). Even today, the clinical significance of positive syphilis test cerebrospinal fluid abnormalities in the absence of neurological clinical findings is unknown (Centre for Disease Control and Prevention, 2024).

14. Even the up-dated technique was unreliable and at risk of false positives and the Wassermann test was later replaced by other more reliable and less technically complicated blood and cerebrospinal fluid tests (Hemarajata, 2020).

15. The potential role of nutritional supplementation in improved outcomes has been discussed by Clayton (2022).

16. Joel Braslow’s research into malaria therapy in the local Californian setting is important to highlight in this respect. Braslow (1997) “intentionally avoided making an argument for or against the efficacy” of malaria therapy and considered that no judgements could be made about this with any degree of certainty due to absence of randomized clinical trials (p. 93). However, he concluded it possible that the doctors and patients shared belief in malaria therapy’s effectiveness and the improved doctor-patient relationship created malaria therapy’s apparent effectiveness, so that this “history could, and probably should be, read not as a triumph of biology but as the triumph of hope, and of a desire to heal and be healed” (p. 94).

17. Interestingly, American physicians, Solomon and Viets (1924), observed that prior to the era of tryparsamide and malaria therapy treatments, many GPI patients could experience improvements for a matter of months and that there were many “in state hospitals who could be back at work, but they are general paralytics and a frequent attitude of staff is that they cannot get better”. However, once a treatment claimed to be effective was implemented “a no more definite improvement” could be “viewed as a striking therapeutic success, and the patient reinstated in the community” (p. 899).

18. In 1931, Ellery left the Lunacy Department altogether to set up in private practice (Kaplan, 2014: 1692).

19. These reports are dated the year after the year to which the report refers. There is no report dated 1929, rather both the report for the year of 1928 (which I have called 1930a) and the report for 1929 (I have called 1930b) were dated 1930. The reports are available at: https://www.vgls.vic.gov.au/client/en_AU/vgls
